# Medical News

**Published:** 1856-03

**Authors:** 


THE MEDICAL EXAMINER.

PHIADLELPHIA, MARCH, 1856.

### MEDICAL NEWS.

We invite the attention of the profession to the following letter upon the much vexed question of assimilated rank in the Navy. They will perceive by it that the General Order upon that subject, passed several years since, whether from apathy or fear in the Department to enforce its observance or not we do not know, is entirely set at nought and disregarded in certain quarters. And what is even worse, that the letter of the regulation, or rather a far-fetched and quibbling interpretation of a certain clause in it, is taken advantage of by officers commanding our national vessels to refuse the Surgeons of them the usual privileges and courtesies granted to every commissioned officer of the line. We cannot say positively whether the case presented is or is not a solitary instance of usurpation of power in one, dressed in a little brief authority,' though we have reason to think that it is not; if the same reluctance, however, to allow a respectable position to our Surgeons should be found to exist generally in the navy, the result must be that many will be deterred from entering so intolerant a service.

The profession has previously taken much interest in this matter; it is greatly owing, indeed, to their exertions, that the present regulation was carried through Congress, and we hope they will now use their best efforts, individually and collectively, to have the law so amended as will effectually secure the objects for which it was framed, a law that can be so easily evaded is worse than no law.

### ASSIMILATED RANK OF MEDICAL OFFICERS IN THE NAVY.

Extract of a Letter from a Surgeon to an Officer of the Line of the Navy.

In a note by last mail I said I was willing to forget the past of the cruise. There is a subject, however, upon which I would have freely spoken with you after you were detached from the ship, had a proper occasion offered. It is that of the assimilated rank of medical officers. I design stating to you now very frankly, but yet in a spirit of unalloyed kindness, my notions on this much vexed question. At the time I joined the ship, it was stated to me that you had declared your intention "to be down upon the doctors and pursers," and believing this statement, I inferred that your prejudices were so strong on the subject, you would not patiently listen to views or arguments opposite to your own. I confess that this was to me a source of great discomfort, and perhaps of discontent, which I am not aware, however, of having manifested on any occasion. I felt and still feel that, though a regulation or general order now legalized, had given me a position in the naval community, that no regard whatever was paid to it, and that I am left with less consideration than a citizen, a gentleman who might be a passenger in the ship. The consequence of this was to almost entirely deprive me of all feeling of interest in her. The feeling was enhanced by the constant reference to orders and regulations having connection with other points, and contrasting my recollections of my former associations afloat. I felt that after nearly thirty years in the navy, of which nearly a quarter of a century had passed over my head as a surgeon, that for the third time I was honored with the appointment of Surgeon of the Fleet, feeling too that I have some professional reputation, and that I am no longer a young man, my self-esteem was deeply wounded to find myself placed by the authorities of the ship on a level with the junior assistant surgeon, so far as the acknowledgments of rights, at least as far as such acknowledgment could be made by military manifestations of consideration or respect, or by the internal regulations of the ship. Any gentleman visiting the ship was free to visit the poop, and because I held a commission and responsible appointment under it, I was forbidden to touch that part of the vessel. To commissioned officers of the line it was free. I do not pretend to assign or question the motive. I mention the fact to speak of its influences upon me, and in illustration of the mortification which I everywhero met on *official* points. My constant wish was to be absent from the ship, and see as little of her as possible. I came to feel that I had neither rights nor interests in her.

I felt too that I was without means of asserting what I deemed to be my rights, because the authorities on board had prejudiced and determined in the premises and would not calmly listen to what I might have to urge. In this respect, I regarded the conduct of my superiors as tyrannous, and at their hands I had nothing to expect. Submission was, therefore, my only course, unless I was prepared to quarrel, on a question which, like most others, has two sides, with those who had the power as well as the disposition to render me uncomfortable to a degree which might not be tolerable by a sensitive man. In this frame of mind I contemplated—and I have not yet entirely abandoned the idea—presenting charges against the Commodore, which I believe can be maintained before any tribunal of disinterested and unprejudiced men. But such a Court cannot be found, I fear, in the navy. The prejudices of the Line on the question seem to me too strong to expect such a Court, yet I sometimes fancy that it should be attempted.

The charges would be in brief, "Conduct unbecoming an officer." He refused to obey the General Order, now made law by an act of Congress, which assigns rank to medical officers. He declared he would rather stand a court-martial than give any order touching the rank of medical officers—that he would not obey any order of the Chief of the Bureau of Medicine—declarations unbecoming an officer, commanding in chief, because they suggest insubordination, and are calculated to bring into disrespect the General Orders of the Secretary of the Navy, and the act of Congress creating the Bureaus. 2. Disobedience of Orders. He refused to obey a legalized order of the Secretary of the Navy, by not fully recognizing under it the rank of the surgeon of the fleet, and refused to give the order any construction whatever in practice, on the ground that he did not understand it. By a quibble he argued that because that officer was incidentally surgeon of the flag ship, he could not be granted his rights as surgeon of the fleet. As well might he declare that because that officer is a citizen of Philadelphia, he could not enjoy the rights or privileges which pertain to an officer of the Federal government. 3d. "Oppression." He deprived the Surgeon of the Fleet of the privileges of his rank, and thus degraded him to the level of assistant surgeons. To wilfully deprive a man of his legal rights is both tyrannous and oppressive.

To refuse to obey a legal General Order is disobedience of orders.

To disobey a lawful order is conduct unbecoming an officer.

To degrade an officer in the estimation of his associates, and of his special subordinates, by withholding all the customary signs of recognition of his rank, constitutes oppression, or at least oppressive conduct, and tends to the destruction of military discipline.

I do not think that a man of your grasp of intelligence, high tone, and knowledge, will gainsay these propositions ; and I hope that reflection and calm consideration will bring you to believe that the principles which they embody *should* be applied in the case of a medical officer, as free from bias as they *would* be in the case of an officer of the line. But I will not attempt to argue the matter; my object is simply to give you my opinions, requesting that before you condemn them, you will, in imagination, change positions. Fancy yourself an old surgeon for a while, that you may view the question from my position, and not that of an officer of the line. My theory is, that *gentlemen* may differ widely in opinion, and still be friends, or at least agree not to disagree or quarrel.

Let us look at the law. The General Order, in question, assigns four degrees of rank to medical officers; namely, 1, with Commanders; 2, with Lieutenants; 3, After Lieutenants; 4, After Masters.

The General Order surely designed that these four degrees of assimilated rank should be distinct, and distinguished from each other respectively, just in the same manner, according to naval custom, as the grades of Commander, Lieutenant and Master are distinguished. And, as a manifestation, in part, at least, of these several degrees of rank, a uniform dress, with badges to distinguish them, one from the other, was devised and assigned to medical officers.

My belief is that the assimilated rank was designed to entitle medical officers to the same personal privileges and immunities, the same ceremonials of respect, as are freely enjoyed by officers of all grades of the line with which they are in rank assimilated. My belief is that a Surgeon who holds the assimilated rank of Commander is entitled, while living, to all the courtesies, respect, &c , to which a Commander in the line is entitled, by the customs of the naval service, and when dead, to the same funeral honors. And that a Surgeon, holding assimilated rank with Commanders, cannot be lawfully placed on a level with Lieutenants of the line, or inferior to them, simply for the reason that, according to the customs of the service, a Commander cannot be placed on a level with Lieutenants of the line, or inferior to them, as he would be, were he to be treated ceremonially as a Lieutenant.

My belief is that "precedence" does not, itself, mean or imply command; that is a strained construction of words, which draws such a meaning from it. My belief is that a Lieutenant, acting in the capacity of executive officer or first Lieutenant, has no right, under the law, to control any medical officer with the rank of Commander, in any manner or form; and further, there is nothing in their respective duties, which requires that the executive officer should have such right to control the official or personal acts or movements of a Surgeon, holding the rank of Commander, no matter whether he be Surgeon of the fleet or not. It is enough for all official purposes, that the Surgeon or Surgeons of the fleet should be subordinate to the officer in command of the ship to which he may be attached. It is not necessary, for example, in order that the duties of the medical department of the ship shall be properly discharged, that the first Lieutenant shall have the right to command the Surgeon, or the Surgeon of the fleet, not to leave the ship, or to be informed when he goes or when he comes. He can as well be informed of this by the officer of the deck, as of the ingress and egress of the Captain, Commodore, or his Secretary. The government confides to the medical officer the management of the medical department, and has provided means of punishing for neglect of duty, or for disappointing its confidence in any manner.

I believe that the law which confers assimilated rank, is as binding, on all officers of the navy, as any other law. And to ignore its existence or to transgress it, is disobedience and open disrespect to the will of Congress, and the will of the Executive.

So much for the construction and application of the law as it stands. But my notion is that no medical officer could safely attempt to enforce it on board of this ship, without being assailed by every annoyance which illiberal and tyrannous dispositions might devise. There is, perhaps, an apathy in the N vy Department on this, as well as on other points, and complaints might be received with indifference from physicians ten thousand miles away from the ear of the Secretary of the Navy and the public.

Whether the *law* is just or proper, as a whole or in part, is a question distinct and independent of its application or administration.

I believe the law is defective, and that it would work more harmoniously, and certainly without affording place for quibbles on its meaning and application, if the exceptional clause were stricken out. I mean that clause which gives precedence to Commanding and Executive officers. My belief, too, is that without this clause, the administration of the law could, in no manner whatever, injure the discipline or efficiency of the naval service, the true interest of which I have as much at heart as any man in it. Unless this clause be stricken out, it would be advisable to repeal the whole law, and place medical officers at the discretionary courtesy of officers of the line, in spite of the fact that former experience is loud against it.

I do not fancy that you agree with me in these notions; yet I conjecture that you might find it difficult to demonstrate that my views have no foundation. I have always thought, however, that on shipboard such points should not be discussed, because few men, (according to my experience of the line,) have magnaninity enough, while in power, to yield any point, which, even in appearance, trenches upon it. But I believe that you will, while off duty, consider the subject with the liberal spirit which has often exhibited itself to me in our conversations on various subjects, and perceive that every position I take is, in itself, absolutely true. I wish to extend the truth; nothing more.

---

PHILADELPHIA, February 12th, 1856.

At the last stated meeting of the College of Physicians of Philadelphia, the following delegates were elected to the next meeting of the American Medical Association.

Drs. Franklin Bache, George B. Wood, Rodman Paul, Charles D. Meigs, George W. Norris, John B. Biddle, Robert P. Thomas, Thomas H. Yardley, Alfred Stillé, William V. Keating, William B. Page, and Henry Hartshorne.

ALFRED STILLÉ, Secretary.

---

The Catalogue of the Jefferson Medical College of Philadelphia contains the names of 510 students.

---

Dr. J. Da Costa has been appointed Lecturer on the Theory and Practice of Medicine in the Philadelphia Association for Medical Instruction, the position previously occupied by the late Dr. Moreton Stillé. Dr. Da Costa is a gentleman of excellent abilities and much information, and will prove a valuable addition to the school.

---

Dr. Marshall Hall has had the honor of being elected the Corresponding Member to the section of Medicine and Surgery in the French Institute, receiving 39 out of 41 votes. The list of candidates consisted of himself, Dr. Rokitansky, of Vienna, Prof. Christison, of Edinburgh, M. Riberi, of Turin, and M. Chelius, of Heidelberg.

---

MORTALITY OF NEW YORK AND BROOKLYN IN 1855.—NEW YORK.—The total mortality in the city of New York during the year 1855, was 22,179, which is 6,389 less than in 1854, notwithstanding the supposed increase of population. The total mortality of 1854 was 28,568, of which number 2,509 died of cholera, which prevailed here more or less from June to November. There has been a remarkable exemption from all epidemic diseases during the past year. More than *half* the deaths occurred among those under *five* years of age, which class comprehends a little less than *one-seventh* of the whole population; while in the class from 5 to 20 years, which embraces more than *one-fourth* of the entire population, there were only 1,545 deaths, being one death in 109 individuals. The deaths in the class from 20 to 40 were *one* in 70; in those over 40, *one* in 38. In 1854, the weekly average of deaths was 547; in 1855, it was 426. The population of the city is probably about 650,000.

*Brooklyn.*—The total mortality in Brooklyn during the year 1855, was 3,893, of which number 2,031 were males, and 1,862 females. Of this number, 424 died of consumption, 177 of pneumonia, 154 of croup, 286 of cholera infantum, 239 of scarlet fever, 48 of measles, 9 of small pox, and 61 of typhus and typhoid fevers.—*N. York Medical Times.*

---

Dr. Bennet's Introductory Lecture, which has been published under the title of the "Present State of the Theory and Practice of Medicine," is exciting considerable attention just now, on account of a pamphlet remarking on it in terms of great severity, by Dr. John M'Gilchrist, a gentleman of great talent, and not unknown, we understand, to the frequenters of your London theatres, by some of his performances, which have been acted on the stage.

The criticism of Dr. M'Gilchrist was originally communicated to the Hunterian Medical Society, and has since been published.

The proem which Dr. M'Gilchrist lays down, is stated as follows:—

"It is because I conscientiously differ from Dr. Bennet,—because I consider the scientific position he would assume for modern Medicine unphilosophical and untenable,—no less than because I think his Lecture a narrow and illogical production,—that I embrace this opportunity of indicating what I believe to be some of the specious fallacies—the barren speculations—the *ignes fatui* of modern medicine."

Having thus discussed the questions raised by the Lecture, Dr. M'Gilchrist concludes with the following verdict, not very complimentary to the learned Professor:—

"We have now done with Dr. Bennet's Lecture,—a production, we make bold to say, remarkable for the narrowness of its views, the absurdity of its illustration, and the ignorance it betrays of the first principles of ratiocination."

A pamphlet written in a spirit of such uncompromising boldness could not fail to produce considerable excitement, especially among the

Students, and it forms the constant theme of conversation in Professional circles.

Dr. Bennet has certainly got an ugly customer to deal with, and the general opinion seems to be, that his interest will counsel him to consult the better part of valor—discretion.

The less sympathy is felt for Dr. Bennet under this castigation, on account of the offence which has been given to the Profession by a paper published by him in the current number of the *Monthly Journal*, "On the Diagnosis of Phthisis Pulmonalis."

In this paper, the author somewhat arrogantly, as is thought, assumes a superiority in diagnosis which those who have watched the non-verification of his prognostics, by dissection in the Infirmary, are by no means willing to accord to him. The reaction produced, in consequence of this assumption, has called to mind many stories previously floating about, such as those to which Professor Henderson has given embodiment, in his "Homœopathy fairly Represented."

"I have known," says Dr. Henderson, "a great advocate for Cod-liver oil in consumption, mistake chronic pleurisy for the other disease. I have known an eminent stethoscopist, for mere irritation of the throat, which he treated with caustic as usual, mistake pulmonary consumption, which was fatal within the week by the bursting of a tubercular abscess into the pleura. I have known an instance in which a notable Hospital Physician, not finding, on dissection, the pulmonary disease he had mapped out and described to his pupils, adroitly remarked, 'Gentlemen, you perceive the appearances on dissection don't correspond with the stethoscopic signs heard during life' (the lung was sound)."—*Medical Times and Gazette.*

---

The pregnancy of the Empress of the French is an event scarcely second to any of the present age in importance, not only to the destinies of France, but to the interests of Europe and the world at large. Sinister rumors have been set afloat in Paris by those disaffected to the Bonapartist dynasty; and it is in the highest degree imperative that the gestation of her Imperial majesty, and the birth of a direct heir to the Empire, should receive the highest scientific attention, and be watched with the greatest and most impartial assiduity. We alluded to this subject in the last number of The Lancet; and we are informed on good authority, that Dr. Locock wishes it to be mentioned, that certain of the points referred to by our "Correspondent," are not entirely correct. The Empress was in this country in April last, and we believe Dr. Locock was consulted at that time; and he was subsequently summoned to the

Palace of the Tuileries. The Empress Eugenie did not become pregnant until the last week in June. Dr. Locock was first consulted in this country, and he then attended a consultation of distinguished physicians and surgeons in Paris before that auspicious event occurred. Dr. Locock has not yet been requested to attend at the approaching accouchement of the Consort of Napoleon III., he has not, therefore, declined to attend. There are reasons of State, in France, for and against his attendance, but if his presence should be called for, we do not see why Dr. Locock should refuse his services.—*London Lancet.*

---

The jury impanelled to inquire into the cause of the death of Walter Palmer, have returned the following verdict:—"We find that Walter Palmer died from the effects of prussic acid, and that such prussic acid was wilfully administered by William Palmer." The case presents many features of great interest; but, at this stage of the proceedings, it would be premature to make any observations upon the evidence adduced in support of the finding of the jury.—*Ibid.*

---

M. Müller.—The distinguished physiologist of Berlin only escaped drowning the other day by his presence of mind. He was returning from Norway, when the steamer in which he was came in contact with another vessel and foundered. M. Müller owed his safety to his skill in swimming.—*Edinburgh Medical Journal.*

---

In consequence of the death of M. Valleix, the following changes have taken place in the hospital staff of Paris. M. Becquerel passes from the Hôpital Riboisière to La Pitié, and M. Herard, of the Hôpital Saint Antoine, takes his place. M. Moutard Martin is transferred from the Institute for Nurses to the Hôpital Saint Antoine, and is succeeded by M. Beugeron, of the Hospice de Rochefoucauld, while M. See, of the Bureau Central, takes his place.—*Ibid., from Gaz. Hebdom.*

---

A Dinner of Horseflesh.—M. Renault, director of the Veterinary School of Alfort, has just given to a few medical friends a quaint kind of dinner, in which the merits of horseflesh, as an article of food, were to be compared to those of beef. The repast was partly composed of soup, the boiled meat used for it, and a roasted joint, each respectively and separately prepared, on the one hand with horseflesh, and on the other with meat from the ox. The company (who were, however, presented with many other dishes and delicacies) were unanimously of opinion that the horse had won the palm.—*Ibid.*

